# Effects and mechanisms of pirfenidone, prednisone and acetylcysteine on pulmonary fibrosis in rat idiopathic pulmonary fibrosis models

**DOI:** 10.1080/13880209.2016.1247879

**Published:** 2016-12-09

**Authors:** Wencheng Yu, Fang Guo, Xiaoxia Song

**Affiliations:** a Department of Respiratory Medicine, The Affiliated Hospital of Qingdao University, Qingdao, China;; b Department of Pediatrics, Laiwu City People’s Hospital, Laiwu, China;; c Department of Intensive Care Unit, The Affiliated Hospital of Qingdao University, Qingdao, China

**Keywords:** Caveolin-1, tumor necrosis factor-α, transforming growth factor-β1, platelet derived growth factor, airsacculitis, expression level

## Abstract

**Context:** Previous studies have reported that caveolin-1 (Cav-1) is associated with lung fibrosis. However, the role of Cav-1 expression in pirfenidone-treated idiopathic pulmonary fibrosis (IPF) is unknown.

**Objective:** This study investigated Cav-1 expression in pirfenidone-treated IPF, and compared the effects of pirfenidone with acetylcysteine and prednisone on IPF.

**Materials and methods:** Rat IPF model was established by endotracheal injection of 5 mg/kg bleomycin A5 into the specific pathogen-free Wistar male rats. Pirfenidone (P, 100 mg/kg once daily), prednisone (H, 5 mg/kg once daily) and acetylcysteine (N, 4 mg/kg 3 times per day) were used to treat the rat model by intragastric administration for 45 consecutive days, respectively. The normal rats without IPF were used as the controls. After 15, 30 and 45 days of drug treatment, lung histopathology was assessed. The expression of Cav-1 was determined using real-time quantitative PCR and Western blot; the expression of tumour necrosis factor-α (TNF-α), transforming growth factor-β1 (TGF-β1) and platelet-derived growth factor (PDGF) was determined by enzyme-linked immunosorbent assay.

**Results:** After 15, 30 and 45 days of drug treatment, comparison of the three drug-treated groups with the model group showed significantly lower (*p* < 0.05) significance of airsacculitis and fibrosis scores of lung tissues, as well as expression of TGF-β1, TNF-α and PDGF, but the expression of Cav-1 was higher (*p* < 0.05). Compared with the N group, the fibrosis score was significantly lower and the protein expression of Cav-1 was significantly higher in the P group (*p* < 0.05). Additionally, the expression of Cav-1 was negatively correlated with the airsacculitis and fibrosis scores (*r* = −0.506, *p* < 0.01; *r* = -0.676, *p* < 0.01) as well as expression of TGF-β1, TNF-α and PDGF (*r* = −0.590, *p* < 0.01; *r* = −0.530, *p* < 0.01; *r* = −0.553, *p* < 0.01).

**Discussion and conclusion:** Pirfenidone, prednisone and acetylcysteine can inhibit airsacculitis and pulmonary fibrosis in rat IPF models, which may be related with enhanced caveolin-1, reduced TNF-α, TGF-β1, PDGF.

## Introduction

Idiopathic pulmonary fibrosis (IPF) is the most common idiopathic interstitial pneumonia and characterized by extracellular matrix (ECM) remodelling and abnormal proliferation of fibroblasts in pulmonary parenchyma (Thannickal et al. 2004; Prasse 2015). IPF results from long-term exposure to substances harming alveolar epithelial cells, and also heredity (Prasse 2015). Currently, the pathogenesis of pulmonary fibrosis has not been clearly uncovered, but various cytokines and other molecules have been discovered to play crucial roles in the progression of pulmonary fibrosis, such as tumour necrosis factor-α (TNF-α) (Pantelidis et al. 2001), transforming growth factor-β1 (TGF-β1) (Willis et al. 2005), platelet-derived growth factor (PDGF) (Antoniades et al. 1990) and caveolin-1 (Cav-1) (Shivshankar et al. 2012).

Cav-1 is a key ingredient of the caveolae membrane structures, and it is involved in the regulation of numerous signalling pathways and biological processes (Rothberg et al. 1992; Okamoto et al. 1998). There is evidence that alterations of Cav-1 function lead to ECM production in fibroblasts and are associated with lung fibrosis (Wang et al. 2006; Xia et al. 2010). Cav-1 is markedly downregulated in primary pulmonary fibroblasts and lung tissues from IPF patients, compared with controls (Wang et al. 2006). Furthermore, Cav-1 is able to inhibit the expression of TGF-β1 and PDGF (Yamamoto et al. 1999; Razani et al. 2001), and regulate TNF-α-induced endothelial cell activation and inflammation (Wang et al. 2008). However, in IPF, the relationships of Cav-1 expression and these cytokines are still unknown.

Currently, a number of therapeutic agents for lung fibrosis have been discovered, such as pirfenidone, acetylcysteine and prednisone (Taniguchi et al. 2010; Raghu et al. 2012). Pirfenidoneis has been demonstrated to be a promising agent with therapeutic potential for IPF due to its antifibrotic, anti-inflammatory and antioxidant effects (Gurujeyalakshmi et al. 1999; Iyer et al. 1999; Oku et al. 2008). Studies have reported that pirfenidone is able to inhibit the expression of pulmonary cytokines in murine pulmonary fibrosis, such as TNF-α, TGF-β1 and PDGF (Gurujeyalakshmi et al. 1999; Iyer et al. 1999; Nakazato et al. 2002). However, it is still elusive whether pirfenidone is able to regulate Cav-1 expression in IPF, and Cav-1 expression in the therapeutic agent-treated IPF has not yet been investigated by other studies. Additionally, in the therapeutic agent-treated IPF, the relationships of Cav-1 expression and the cytokines (e.g., TNF-α, TGF-β1, and PDGF) are still unknown. Clarifying these issues contributes to a better understanding of the role of Cav-1 expression in IPF during the agent treatment.

In this study, to investigate Cav-1 expression in IPF after pirfenidone, prednisone and acetylcysteine treatments, and compare the effects of pirfenidone with other two agents (acetylcysteine and prednisone) on IPF, rat IPF model was established by endotracheal injection of bleomycin A5. Expression levels of Cav-1, TNF-α, TGF-β1 and PDGF were determined in the IPF model, respectively, treated by pirfenidone, acetylcysteine, prednisone. Furthermore, correlations of Cav-1 expression with the degree of airsacculitis and fibrosis, as well as expression of TNF-α, TGF-β1 and PDGF were assessed. These results may contribute to a better understanding of the roles of Cav-1 in IPF and the effect of pirfenidone in the therapy of IPF.

## Materials and methods

### Animals and grouping

A total of 75 specific pathogen-free Wistar male rats (200 ± 25 g) were purchased from PaiTeFuDe White Rats Farming Cooperatives of Qingdao city in China (certification number: scxk Lu 20130001). According to the random number table, all rats were divided into five groups randomly: the model group (M), pirfenidone group (P), acetylcysteine group (N), hormone group (H) and control group (C). Each group included 15 rats. All of the rats were fed under pathogen-free conditions and underwent a reversed 12:12 h light/dark cycle. The room temperature was kept constant at 22–25 °C and the relative humidity at 50%. Except for the control animals, all of the animals in other four groups received 5 mg/kg bleomycin A5 by endotracheal injection in order to establish a pulmonary fibrosis model. Starting from the second day after bleomycin injection, rats in the P group were treated with 100 mg/kg pirfenidone once daily, rats in the H group were treated with 5 mg/kg prednisone once daily, rats in the N group were treated with 4 mg/kg acetylcysteine 3 times per day and rats in the M group were treated with 2 mL 0.9% normal saline twice daily. Pirfenidone, prednisone and acetylcysteine were used as the type of aqueous solution or suspension. All drugs were applied using intragastric administration.

After 15, 30 and 45 days of drug treatment, 5 rats in each group were sacrificed using intraperitoneal injection of 10% chloral hydrate method, and 1 mL blood was sampled from the abdominal aorta and then centrifuged. Afterwards, serum samples were stored at −20 °C. Meanwhile, 100 mg left lung tissues of rats were sampled and fixed with 4% formaldehyde solution; 100 mg superior lobe and middle lobe of right lung were respectively sampled, washed clean, cut into pieces and stored at 80 °C. The experiments followed the National Institutes of Health (NIH) guidelines for animal studies. All animal procedures used in the present study were reviewed and approved by the animal ethics committee of Qingdao University.

### Histopathological observation

Left lung tissues having been fixed with 4% formaldehyde solution were embedded in paraffin. Before staining, the sections were incubated at 60 °C for 1 h, dewaxed with xylene and rehydrated through a series of ethanol solutions. Subsequently, sections of lung tissues were stained with hematoxylin and eosin (H&E) [New England Biolabs (Beijing) Ltd., Beijing, China] to observe histological changes in lung tissues of rats under an optical microscope (Olympus CX22; Olympus Corporation, Tokyo, Japan). The degree of airsacculitis and fibrosis was statistically classified according to the Szapiel method (Szapiel et al. 1979).

### Measurements of cytokine levels using enzyme-linked immuno sorbent assay (ELISA)

TGF-β1, TNF-α and PDGF in the serum of rat abdominal aorta were determined using TGF-β1 ELISA kit (catalog number MB100B, R&D Systems, Minneapolis, MN), TNF-α ELISA kit (catalog number DY2279, R&D Systems, USA) and PDGF ELISA kit (catalog number DYC1767-1, R&D Systems, USA), according to the manufacturer’s protocols.

### Determination of cav-1 mRNA level using real-time quantitative PCR (RT-qPCR)

Based on the sequence of Cav-1 in the National Center For Biotechnology Information (NCBI) database, gene primers were designed using the Primer5 software (PRIMER-E Ltd, Plymouth, Devon, UK) as follows: Cav-1-forward primer, 5′-TGTGATTGCGGAACCAGAAGG-3′, reverse primer, 5′-TGCCCAGATGTGCAGGAAAGA-3′; GAPDH-forward primer, 5′-TGGAGTCTACTGGCGTCTT-3′, reverse primer, 5′-TGTCATATTTCTCGTGGTTCA-3′.

Total RNA was isolated from the superior lobe of right lung tissues using an ultrapure RNA extraction kit (CWBio. Co., Ltd., Beijing, China) according to the manufacturer’s instructions. The integrity of RNA was inspected by Agilent Bioanalyzer 2100 (Agilent Technologies, Santa Clara, CA). The eligible RNA was then reverse transcribed into cDNA using a HiFi-MMLV cDNA kit (CWBio. Co., Ltd., Beijing, China). After cDNA synthesis, mRNA expression level of Cav-1 was tested using the Ultra SYBR Mixture (with ROX) (CWBio. Co., Ltd., Beijing, China). Relative gene expression was calculated using the 2^−ΔΔCt^ method (Livak & Schmittgen 2001), and normalized against glyceraldehyde-3-phosphate dehydrogenase (GAPDH).

### Determination of Cav-1 protein level using Western blot

Middle lobe of right lung tissues were lysed in radioimmunoprecipitation assay lysis buffer [l% Triton-100, 0.1% sodium dodecyl sulfate (SDS), 1 mmol/L ethylenediaminetetraacetic acid, 150 mmol/L NaCl, 10 mmol/L Tris-HCl, pH 7.5] for 30 min on ice. Subsequently, the lysate was transferred to Eppendorf tubes, and then centrifuged at 12,000 rpm for 30 min at 4 °C. The supernatant was transferred to a fresh tube and mixed with an equal volume of 2 × SDS and boiled for 20 min. Then, one sample containing 50 μg of protein was fractionated by 10% SDS-polyacrylamide gels (PAGE) and transferred onto polyvinylidene fluoride membrane for 2 h. Following blocking nonspecific binding sites with 5% skimmed milk in 1 × Tris buffer containing 0.1% Tween-20 (TBST, 0.1% Tween 20, 50 mmol/L of Tris, 150 mmol/L of NaCl, pH 7.6) for 1 h, membranes were probed with rabbit anti-mouse Cav-1 monoclonal antibody (dilution 1:1000, catalog number BS1044, Bioworld Technology, St Louis Park, MN) at 4 °C overnight, washed five times with 1 × TBST and incubated with biotin-conjugated secondary antibody (dilution 1:1000, catalog number CW0239, Bio-High Technology, Hebei, China) for 1 h at room temperature. The membrane was visualized with a UVP Bioimaging system EC3 apparatus (UVP, Upland, CA). Finally, the protein expression level of Cav-1 was quantified by image densitometry, and the ratio of Cav-1/β-actin signal was statistically analyzed.

### Statistical analysis

Data were presented as mean ± standard deviation from three separate experiments performed in duplicate. Statistical analysis was performed using SPSS 17.0 software (SPSS, Chicago, IL). One-way analysis of variance was used to compare group means. Differences were considered significant if *p* < 0.05. In addition, correlations of Cav-1 expression and airsacculitis and fibrosis scores as well as cytokines expression were analyzed using the Spearman rank correlation coefficient, and differences among groups were considered significant if *p* < 0.05.

## Results

### General situation of rats

In the C group, rats were active and had good diet and spirit; their fur was smooth and bright; body weight was increasing. However, in the M group, both activity and food intake of rats were reduced; their fur was dark and gloomy; body weight was decreased; furthermore, the rats arched the back and had the cyanosis in the outer edges of limbs and lips, as well as cough and shortness of breath symptoms; before 15 days, hemorrhagic secretion was observed in the nose and mouth of rats and then gradually disappeared. One dead rat in the M group was removed in the statistical analysis. Additionally, the situation of rats in the P, H and N groups was better than that in the M group.

### Assessment of lung histopathology

**Figure 1.**
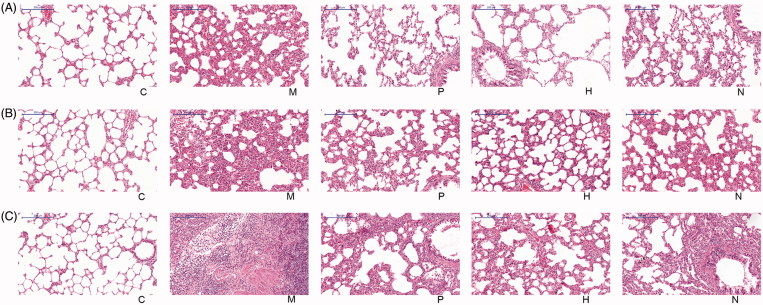
Histopathological images of rat left lung tissues: (a) 15 days after drug treatment. (b) 30 days after drug treatment. (c) 45 days after drug treatment. C, the control group that is not induced into idiopathic pulmonary fibrosis; M, the model group that has been induced into idiopathic pulmonary fibrosis; P, the idiopathic pulmonary fibrosis model group that is treated by pirfenidone; H, the idiopathic pulmonary fibrosis model group that is treated by prednisone; N, the idiopathic pulmonary fibrosis model group that is treated by acetylcysteine. The scale bars were 200 μm.

According to the HE staining, rats in the C group had normal alveolar structure. In the M group, obvious inflammation cell infiltration was observed in the alveolar space and alveolar interval of rats, and the degree of fibrosis was light at 15 days (Figure 1(a)). At 30 and 45 days, alveolar walls became thickened, and a large number of fibroblasts were visible in the alveolar interval; alveolar structure disordered; alveolar space was narrowed and fused; the degree of fibrosis was worse (Figure 1(b,c)). The degree of airsacculitis and fibrosis in the P, H and N groups was obviously lighter than that in the M group at the three time points (Figure 1(a–c)).

Based on the Szapiel method, the degree of airsacculitis and fibrosis was statistically classified. Both the airsacculitis score and fibrosis score in the M group were significantly higher than those in the C group at the three time points (*p* < 0.01). Furthermore, both the airsacculitis score and fibrosis score in the P, H and N groups were significantly lower than those in the M group at the three time points (*p* < 0.05) (Table 1 and 2). The airsacculitis score in the P group was higher than that in the H group, but lower than that in the N group; the difference between P and H as well as P and N was not significant (*p* > 0.05) (Table 1). Additionally, the fibrosis score in the P group was lower at 30 days, but higher at 45 days than that in the H group. However, the difference was not significant (*p* > 0.05). Comparing the P and N groups, the fibrosis score in the P group was significantly lower than that in the N group at the three time points (*p* < 0.05) (Table 2).

**Table 1.** The airsacculitis score of lung tissues in different groups.

Group	15 d	30 d	45 d
C	0.20 ± 0.45	0.00	0.20 ± 0.45
M	2.60 ± 0.55^a^	2.50 ± 0.58^a^	2.20 ± 0.84^a^
P	1.40 ± 0.55^c^^d^^e^	1.20 ± 0.45^c^^d^^e^	1.00 ± 0.71^c^^d^^e^
H	1.20 ± 0.45^c^	1.00 ± 0.00^c^	0.80 ± 0.45^c^
N	1.80 ± 0.45^b^	1.60 ± 0.55^c^	1.20 ± 0.45^c^

Data are presented as mean ± standard deviation. C, the control group that is not induced into idiopathic pulmonary fibrosis; M, the model group that has been induced into idiopathic pulmonary fibrosis; P, the idiopathic pulmonary fibrosis model group that is treated by pirfenidone; H, the idiopathic pulmonary fibrosis model group that is treated byprednisone; N, the idiopathic pulmonary fibrosis model group that is treated byacetylcysteine.

a *p* < 0.01, compared with the C group.

b *p* < 0.05, compared with the M group.

c *p* < 0.01, compared with the M group.

d *p* > 0.05, compared with the H group.

e *p* > 0.05, compared with the N group.

**Table 2.** The fibrosis score of lung tissues in different groups.

Group	15 d	30 d	45 d
C	0.00	0.00	0.00
M	2.80 ± 0.45^a^	2.50 ± 0.58^a^	2.60 ± 0.55^a^
P	1.00 ± 0.71^c^^d^^e^	1.00 ± 0.00^c^^d^^e^	1.40 ± 0.55^c^^d^^e^
H	1.00 ± 0.05^c^	1.20 ± 0.45^c^	1.20 ± 0.45^c^
N	1.60 ± 0.00^c^	2.00 ± 0.00^b^	1.80 ± 0.45^b^

Data are presented as mean ± standard deviation. C, the control group that is not induced into idiopathic pulmonary fibrosis; M, the model group that has been induced into idiopathic pulmonary fibrosis; P, the idiopathic pulmonary fibrosis model group that is treated by pirfenidone; H, the idiopathic pulmonary fibrosis model group that is treated by prednisone; N, the idiopathic pulmonary fibrosis model group that is treated byacetylcysteine.

a *P* < 0.01, compared with the C group;.

b *p* < 0.05, compared with the M group.

c *p* < 0.01, compared with the M group.

d *p* > 0.05, compared with the H group.

e *p* < 0.05, compared with the N group.

### Expression level of Cav-1

**Figure 2.**
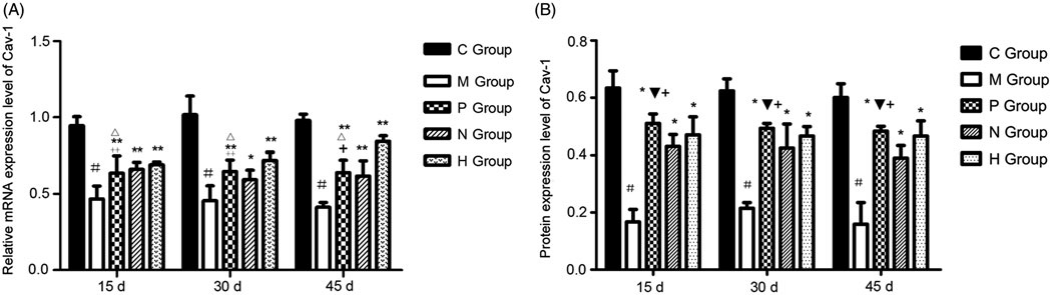
Expression level of caveolin-1 (Cav-1) in rat lung tissues. (a) Relative mRNA expression level of Cav-1 determined by real-time quantitative PCR. #, *P* < 0.01, compared with the C group; **, *P* < 0.01, compared with the M group; *, *P* < 0.05, compared with the M group; +, *P* < 0.01, compared with the H group; ++, *P* > 0.05, compared with the H group; Δ, *P* > 0.05, compared with the N group. (b) Protein expression level of Cav-1 determined by Western blot. #, *P* < 0.01, compared with the C group; *, *P* < 0.01, compared with the M group; +, *P* > 0.05, compared with the H group; ▼, *P* < 0.05, compared with the N group. C, the control group that is not induced into idiopathic pulmonary fibrosis; M, the model group that has been induced into idiopathic pulmonary fibrosis; P, the idiopathic pulmonary fibrosis model group that is treated by pirfenidone; H, the idiopathic pulmonary fibrosis model group that is treated by prednisone; N, the idiopathic pulmonary fibrosis model group that is treated by acetylcysteine..

According to the results of RT-qPCR, both mRNA and protein expression levels of Cav-1 in the C group were higher than those in other four groups. Both mRNA and protein expression levels of Cav-1 in the M group were significantly lower than those in the C group (*p* < 0.01), as well as the P, H and N groups (*p* < 0.05) (Figure 2(a,b)). Comparing the P and H groups, there was no significant difference in the mRNA expression level of Cav-1 between the P and H groups at 15 and 30 days (*p* > 0.05), whereas mRNA expression level of Cav-1 in the H group was significantly higher than that in the P group at 45 days (*p* < 0.01). Furthermore, there was no significant difference in the mRNA expression level of Cav-1 between the P and N groups at the three time points (*p* > 0.05) (Figure 2(a)). Additionally, the difference in the protein expression level of Cav-1 between the P and H groups at the three time points was not significant (*p* > 0.05). The protein expression level of Cav-1 in the P group was significantly higher than that in the N group at the three time points (*p* < 0.05) (Figure 2(b)).

### Correlation of Cav-1 expression with airsacculitis and fibrosis scores

**Figure 3.**
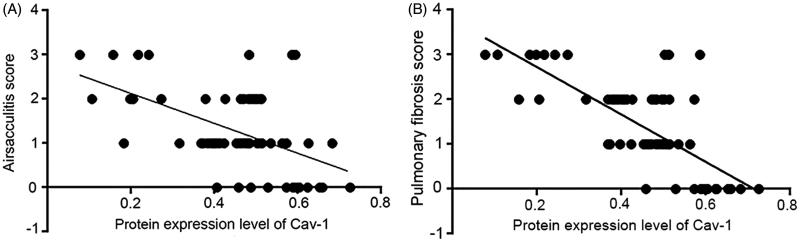
Correlations of caveolin-1 (Cav-1) expression with (a) airsacculitis and (b) pulmonary fibrosis scores in rat.

Both airsacculitis score and fibrosis score were negatively correlated with the protein expression level of Cav-1 (*r* = −0.506, *p* < 0.01; *r* = −0.676, *p* < 0.01). With the increased protein expression level of Cav-1, airsacculitis score and fibrosis score showed a decreased trend (Figure 3(a,b)).

### Expression levels of cytokines in rat abdominal aorta

To evaluate the variation of cytokines in different agent treatments, expression levels of TGF-β1, TNF-α and PDGF in the serum of rat abdominal aorta were determined by ELISA. Expression levels of TGF-β1, TNF-α and PDGF in the M group were all significantly higher than those in the C group, but lower than those in the P, H and N groups at the three time points (*p* < 0.01) (Table 3).

**Table 3.** The expression of cytokines in rat abdominal aorta.

	TGF-β1			TNF-α			PDGF		
Group	15 d	30 d	45 d	15 d	30 d	45 d	15 d	30 d	45 d
C	133.43 ± 4.38	134.59 ± 4.78	134.91 ± 3.76	148.61 ± 5.61	149.84 ± 5.19	151.18 ± 3.73	8.80 ± 0.46	9.65 ± 0.56	9.48 ± 0.41
M^a^	213.11 ± 8.24	205.11 ± 13.85	221.40 ± 10.49	280.06 ± 15.85	277.21 ± 18.89	289.67 ± 12.06	14.53 ± 0.99	14.92 ± 0.65	14.21 ± 0.71
P^b^	148.63 ± 8.63	147.13 ± 5.95	136.96 ± 7.92	191.28 ± 19.06	177.94 ± 8.55	185.88 ± 11.92	11.19 ± 0.67	10.79 ± 0.74	10.52 ± 0.45
H^b^	142.22 ± 4.70	137.50 ± 2.70	134.91 ± 2.82	159.32 ± 10.18	156.99 ± 12.83	155.29 ± 12.28	0.68 ± 0.73	9.73 ± 1.04	9.75 ± 0.95
N^b^	175.41 ± 15.03	166.36 ± 10.28	164.43 ± 24.14	200.80 ± 5.81	191.60 ± 6.01	179.12 ± 14.29	11.30 ± 0.80	10.79 ± 0.86	11.03 ± 0.83

Data are presented as mean ± standard deviation. C, the control group that is not induced into idiopathic pulmonary fibrosis; M, the model group that has been induced into idiopathic pulmonary fibrosis; P, the idiopathic pulmonary fibrosis model group that is treated by pirfenidone; H, the idiopathic pulmonary fibrosis model group that is treated by prednisone; N, the idiopathic pulmonary fibrosis model group that is treated by acetylcysteine.

a *p* < 0.01, compared with the C group.

b *p* < 0.01, compared with the M group.

### Correlation of Cav-1 expression with cytokines

**Figure 4.**
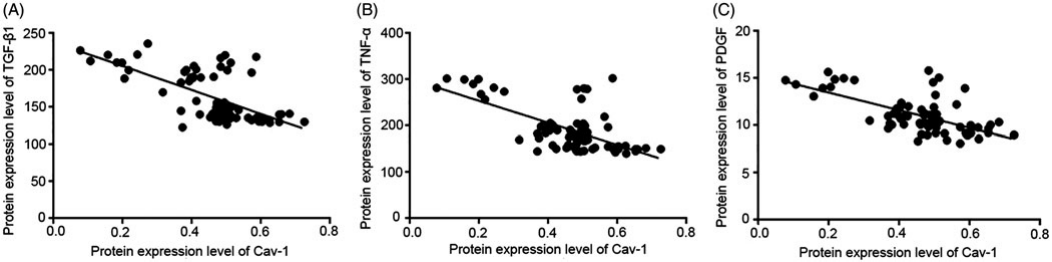
Correlations of caveolin-1 (Cav-1) expression with cytokines in rat. (a) Correlation of Cav-1 with transforming growth factor-β1 (TGF-β1) expression. (b) Correlation of Cav-1 with tumor necrosis factor-α (TNF-α) expression. (c) Correlation of Cav-1 with platelet derived growth factor (PDGF) expression.

The expression levels of TGF-β1, TNF-α and PDGF were all negatively correlated with the protein expression level of Cav-1 (*r* = −0.590, *p* < 0.01; *r* = −0.530, *p* < 0.01; *r* = −0.553, *p* < 0.01). With the increased protein expression level of Cav-1, the three kinds of cytokines showed a decreased trend (Figure 4(a–c)).

## Discussion

In the present study, rat IPF model was established by endotracheal injection of bleomycin A5 in order to investigate the role of Cav-1 expression in IPF after pirfenidone, acetylcysteine and prednisone treatments, and compare the effects of pirfenidone with acetylcysteine and prednisone on IPF.

According to HE staining, both airsacculitis score and fibrosis score of lung tissues in the P, H and N groups were significantly lower than those in the M group at the three time points (*p* < 0.05), indicating that pirfenidone, acetylcysteine and prednisone were able to alleviate the symptoms of airsacculitis and fibrosis of IPF. The three kinds of drugs have been used in the clinical therapy of IPF (Taniguchi et al. 2010; Raghu et al. 2012). In this study, mRNA and protein expression levels of Cav-1 in the P, H and N groups were significantly higher than those in the M group (*p* < 0.05), and the expression levels of TGF-β1, TNF-α and PDGF were significantly lower than those in the M group. These results suggested that pirfenidone, acetylcysteine, and prednisone were able to increase the Cav-1 expression and reduce the expression of TGF-β1, TNF-α and PDGF. Cav-1 is considered as a critical regulator of lung fibrosis in IPF (Wang et al. 2006). A previous study has reported that Cav-1 is significantly downregulated in lung tissues from IPF patients compared with controls, and Cav-1 distinctly relieved bleomycin-induced pulmonary fibrosis (Wang et al. 2006), which was consistent with the results of lower Cav-1 expression in the M group compared with the C group as well as the negative correlation between Cav-1 expression and airsacculitis score and fibrosis score in this study. In bleomycin-treated mice, Cav-1 expression in leucocytes is reduced, which contributes to fibrotic lung disease (Tourkina et al. 2010). Similarly, Cav1-knockout mice were discovered to develop pulmonary and skin fibrosis, and restoration of Cav-1 function *in vitro* normalized the phenotype and abrogated TGFβ activation (Galdo et al. 2008). There is evidence that TGF-β1 is able to inhibit the Cav-1 expression in human pulmonary fibrosis (Lasky & Brody 2000), which supports the result of the negative correlation between Cav-1 and TGF-β1 expression in this study. Furthermore, Cav-1 is an inhibitor of PDGF proliferative responses, and PDGF is also able to result in the loss of Cav-1 expression (Peterson et al. 2003), which is consistent with the result of the negative correlation between Cav-1 and PDGF expression in this study. In addition, in this study, TNF-α expression was also found to be negatively correlated with Cav-1 expression. A previous study has discovered that TNF-α expression leads to the reduced Cav-1 expression in vascular endothelial cells (Sun 2003), and Cav-1 regulates TNF-α-induced endothelial cell activation and inflammation (Wang et al. 2008). TNF-α also inhibits Cav-1 expression in alveolar macrophages (Fakhrzadeh et al. 2008). These studies supported the results of this study. Taken together, in the lung of IPF treated by pirfenidone, acetylcysteine and prednisone, increased expression of Cav-1 may contribute to the remission of airsacculitis and fibrosis through its negative correlations with TGF-β1, TNF-α and PDGF.

In this study, comparing the P and H groups, there were no significant differences in the airsacculitis score and fibrosis score, as well as the protein expression level of Cav-1 between the two groups, indicating the similar effects of pirfenidone and prednisone on airsacculitis and pulmonary fibrosis as well as the Cav-1 expression. A previous double-blind, multicenter study has showed that the efficacy between pirfenidone and prednisone in the therapy of IPF was similar (Moises et al. 2003), which supports the results of this study. Furthermore, comparing the P and N groups, the fibrosis score in the P group was significantly lower than that in the N group, and the protein expression level of Cav-1 in the P group was significantly higher than that in the N group at the three time points, indicating the better efficacy of pirfenidone than acetylcysteine in the pulmonary fibrosis of IPF. Currently, there are no other studies to compare the efficacy of pirfenidone with acetylcysteine in the therapy of IPF. Therefore, pirfenidone is a promising drug for the therapy of IPF, and its efficacy may be better than other drugs like acetylcysteine.

In conclusion, pirfenidone, prednisone and acetylcysteine can inhibit airsacculitis and pulmonary fibrosis in rat IPF models, and its mechanisms may be related with enhanced caveolin-1, reduced TNF-α, TGF-β1, PDGF. Efficacy of pirfenidone in airsacculitis and pulmonary fibrosis was similar to prednisone, while better than acetylcysteine. These findings may be helpful to a deeper understanding of the role of Cav-1 in IPF and the effect of pirfenidone in the therapy of IPF.
